# Supplementary material for: Are carbon emissions trading and green financial instruments synergistic? -Comprehensive quantitative research based on content analysis
Source: PLoS One. 2024 Mar 7;19(3):e0298601. doi: 10.1371/journal.pone.0298601 (PMC10919601; doi:10.1371/journal.pone.0298601)
Supplement: S1 Data — (ZIP) [file pone.0298601.s001.zip › Data/地级市Z_F工作报告.zip!/║╙─╧/╨φ▓2╩╨/└┤╫╘╣1⁄2╓┌║┼í╛┬φ┐╦╩2╛▌═°í┐_2006.docx]

各位代表：

现在，我代表市人民政府，向大会作工作报告，请予审议，并请各位政协委员和列席人员提出意见。

一、2005年工作回顾

2005年，市政府在省委、省政府和市委的正确领导下，开拓进取，扎实工作，圆满完成了市四届人大六次会议确定的各项目标任务。全年完成生产总值603亿元，增长13％。地方财政一般预算收入19.96亿元，增长29.4％。城镇登记失业率控制在3％以内，人口自然增长率4.26‰。经济社会各项事业取得显著成就，在全面建设小康社会实践中迈出了坚实步伐。

项目建设和招商引资成效显著。全社会固定资产投资完成239亿元，增长47.2％，其中城镇以上固定资产投资完成165亿元，增长49.1％。年初确定的90个重点项目完成投资69.8亿元，其中许昌卷烟总厂技改、黄河集团新建金刚石生产线、许昌至尉氏高速公路等28个项目已经竣工。全年招商引资90.8亿元，增长82％。新批准设立外商投资企业31家；实际利用外资4260万美元，增长34.9％。

经济结构进一步优化。二、三产业比重达到83.5％，比上年提高0.7个百分点。规模以上工业完成增加值208亿元，增长22.1%；工业对经济增长的贡献率达71.2%；高新技术产业增加值比上年增长63%。“一城三基地”建设成效明显，国家（许昌）电力电子系统产业园获得批准。特色农业规模不断扩大，优质小麦、花木、中药材面积分别达到140万亩、51.1万亩和35.5万亩。畜牧业增加值占农业增加值的比重达36.7％。农业产业化经营取得新进展，20家重点农业龙头企业完成销售收入34.4亿元，众品公司在美国纳斯达克成功上市。农业科技园区规划实施项目46个，完成投资2.1亿元。旅游、房地产、物流等第三产业快速发展。非公有制经济规模和质量不断提升，完成增加值347亿元，增长20.1%。

改革开放迈出新步伐。国有（集体）企业改革步伐加快。粮食流通体制改革取得突破性进展。农村信用社改革成效明显。乡镇机构改革按时完成。行政审批制度改革不断完善。财政管理体制、投资体制改革稳步推进。事业单位三项制度改革不断深化，36家产权制度改革已经完成。市场经济秩序日益规范，发展环境进一步优化。对外开放不断扩大，进出口总额4.2亿美元，增长13.7％，其中出口3.6亿美元，增长 14%。

经营城市和创建工作取得新进展。城市总体规划和区域性规划获得省政府批准。成功发行两期城建信托计划，融集城建资金3.7亿元。市区24项城建重点工程完成投资9.5亿元。深入开展“‘创三城’和实施碧水蓝天工程”活动，城乡环境质量进一步提高，人居环境明显改善，荣获“国家园林城市”称号。

城乡居民生活水平稳步提高。农业税全免、粮食直补、良种补贴、农机具补贴等政策得到全面落实。新建、改建农村公路526公里。建设农村饮水安全工程，解决了1.6万人的饮水安全问题。减少农村贫困人口1.5万人。下岗失业人员实现再就业2万人，城镇新增就业人员5.9万人。养老、医疗、失业、工伤、生育保险和城乡低保、农村五保供养制度不断完善。城镇居民人均可支配收入7769元，增长11.6%；农民人均纯收入3643元，增长12.2%。

科教文卫等事业协调发展。实施省级以上科技项目65项，获得省级以上科技进步奖10项，申请专利457件，科技进步对经济增长的贡献率达49%。“普九”成果得到巩固提高。农村中小学D级危房改造三年目标提前完成。对农村义务教育阶段贫困家庭学生实施“两免一补”。职业教育工作获得国务院表彰。新型农村合作医疗试点工作扎实推进。人才队伍建设取得新成果。广播电视和文化、体育事业繁荣发展。人口和计划生育、国土资源、环境保护工作进一步加强。“双拥”和军民共建活动深入开展。民族宗教工作成绩显著，被国务院授予“全国民族团结进步模范集体”。统计、审计、外事、侨务、气象、地震、人防、史志、档案等工作都取得了新成绩。

精神文明和民主法制建设得到加强。保持共产党员先进性教育活动扎实有效，精神文明建设取得新的成绩。自觉接受人大法律监督、工作监督和政协民主监督，办理人大代表议案、建议94件、政协委员提案181件。认真落实《依法行政实施纲要》，政府法制建设进一步加强。第五届村民委员会换届选举顺利完成，社区建设取得较大突破。坚持依法治市，全民法律意识明显增强。加强社会治安综合治理，依法严厉打击各种违法犯罪活动。高度重视人民群众来信来访，社会大局保持稳定。

各位代表，上述成绩的取得，是省委、省政府和市委正确领导的结果，是市人大、市政协监督、支持的结果，是全市人民共同奋斗的结果。在此，我代表市人民政府，向工作在全市各条战线的工人、农民、知识分子、干部、人民解放军指战员、武警官兵以及各界人士致以崇高的敬意！向所有关心支持我市现代化建设的同志们、朋友们表示衷心的感谢！

在肯定成绩的同时，我们也清醒地看到，我市经济社会发展中还存在着一些困难和问题。主要表现在：中心城市带动能力较弱，城镇化水平不高；产业结构不尽合理，服务业发展相对滞后；出口产品结构单一，利用外资规模较小；安全生产、资源约束问题比较突出；政府部门作风和效能建设有待进一步增强等。对此，我们一定高度重视，在今后的工作中认真加以解决。

二、2006年的主要目标和重点工作

2006年是实施“十一五”规划的起步之年。我市经济社会发展的主要

目标是：生产总值增长13％；工业增加值增长16％；全社会固定资产投资增长25％，其中城镇以上固定资产投资增长28％；社会消费品零售总额增长13％；进出口总额增长16％，其中出口总额增长18％；地方财政一般预算收入增长18％；农民人均纯收入增长6％，城镇居民人均可支配收入增长9％；人口自然增长率控制在5‰以内；城镇新增就业4.4万人，城镇登记失业率控制在4％以内；万元生产总值能耗下降4％左右。

围绕上述目标，今年重点抓好以下8个方面的工作：

（一）强力推进项目建设和招商引资

加强重点项目建设。今年，全市共安排重点项目100个，其中续建、新建项目86个，前期工作项目14个，年度计划投资100亿元。工业方面，着力抓好东城区热电厂、烟草薄片等53个重点项目，当年完成投资50亿元，争取30个项目建成投产。农林水方面，继续推进国家农业科技园区、市区拦蓄水工程建设，加快优质小麦基地、花卉苗木基地、中药材基地、生猪生产和加工基地建设，搞好农业产业化和信息化项目建设。交通方面，加快许禹、许亳、禹登及郑石高速许昌段和国（省）道许昌段改扩建工程建设。积极推进禹登铁路、许禹铁路建设以及郑州至武汉客运专线许昌段的前期工作。社会事业方面，加快许昌学院二期、许昌职业技术学院迁建、许昌二高迁建、数字电视客户服务中心等项目建设。要继续把重点项目纳入目标管理，完善重点项目市级领导联系制、项目法人责任制、招投标制、工程监理制、合同管理制和竣工验收制等工作机制，切实解决项目核准、资金落实、征地拆迁、建设环境等问题，形成合理的投资规模和良好的投资环境。

加大招商引资力度。抓住国家推进社会主义新农村建设、促进中部地区崛起战略实施、发行国债资金和省里增加安排专项资金的机遇，努力争取更多的发展资金。加强招商队伍建设，创新招商方式，有针对性地开展招商活动。加强对签约项目的跟踪问效，确保合同兑现、资金到位。认真落实企业招商引资奖励政策，提高企业招商引资积极性。优化金融生态环境，推动银企合作，继续搞好与驻郑州各股份制银行的对接和开行授信工作，积极推进企业发行股票、债券，提高企业的直接融资能力。

（二）扎实推进社会主义新农村建设

认真抓好新农村示范区建设。围绕“生产发展、生活宽裕、乡风文明、村容整洁、管理民主”的总目标，按照“统一规划、市场运作、产业支撑、农民受益”的要求，抓好许昌至长葛87平方公里区域内城乡一体化、新农村示范区建设。前期重点规划和建设“一轴、二区、三园、三地和61村”，即以许昌至长葛城际通道为轴线，以许昌县新区和长葛市新区为连接，以河南（魏都）民营科技园、长葛市工业物流园、许昌县尚集民营工业园为产业支撑，以长葛市生态园、许昌县生态园、魏都区生态园为生态绿地，与分散布局的61个行政村协调发展的社会主义新农村示范区。各县（市）都要选好试点，合理布局，科学规划，使社会主义新农村建设整体推进、协调发展。

积极推进现代农业建设。稳定提高粮食生产能力，推广高产优质品种及配套技术。积极发展特色农业，新增花木8.5万亩、木本中药材3万亩、退耕还林3.3万亩。巩固提高311国道许昌至鄢陵段、107国道许昌至长葛段花卉长廊建设的规模和档次，积极推进颍汝百里林水长廊、鄢陵花木市场和兆丰花卉市场建设。投资3360万元，建设烟叶现代园区。抓住省政府发展“郑许漯”食品带机遇，大力发展食品加工业。积极扶持20家重点农业龙头企业加快发展，引导龙头企业直接融资，建立原料基地，搞好农产品精深加工。抓好“千场百区”建设，大力发展养殖业。加快农业信息化步伐，实施“农村科技示范工程”。推进农业机械化，提高劳动生产率。积极推进土地流转，提高集约经营水平。

千方百计增加农民收入。继续实施“五型”增收，使农民群众不断得到实惠。培训农村富余劳动力15万人，新增转移、输出农村富余劳动力20万人次。加大扶贫开发力度，解决1.5万农村贫困人口脱贫问题。切实加强涉农收费及价格管理，防止农民负担反弹。不折不扣地落实好对种粮农民直接补贴、农业生产资料综合直补、良种补贴、大型农机具购置补贴等七项惠农政策，做到“一分钱也不能少，一天也不能耽误，一户也不能漏掉”。

切实加强农村基础设施建设。以“饮干净水、走平坦路、建沼气池、用卫生厕”为目标，实施农村饮水安全工程，解决33个行政村、4万人的饮水安全问题；投资2.6亿元，改造、建设农村公路600公里；积极推广使用沼气，力争新增沼气用户1万户；综合整治村容村貌，逐步建立长效机制。加快农村电网改造的续建配套工程。搞好农业综合开发和农田水利基本建设。推进“万村千乡市场工程”，建设连锁化“农家店”。完善县级农产品、畜产品质量检测检验体系和禽流感等重大动物疫病防控体系。

（三）坚定不移地推进工业化

积极推动工业结构优化升级。围绕“136”工业体系建设，启动实施“技术创新引导工程”，抓好一批工业重点技改项目，推动传统产业改造提升和高新技术产业发展。继续采取规划引导、要素倾斜、政策扶持等措施，增强支柱产业、重点企业的支撑带动作用。着力抓好省、市制造业信息化重点示范企业，提升我市制造业信息化水平。强力推进国家（许昌）电力电子系统产业园建设，切实抓好许昌经济技术开发区和河南（魏都）民营科技园区等一批有优势的产业带、工业园区的建设和发展，提高产业集聚度。继续开展“质量立市、名牌兴业”活动，引导企业培育品牌、提升品牌、经营品牌。

加快重点企业发展。认真落实支持、扶持重点企业发展的各项政策。鼓励、引导重点企业加大研发投入，提高自主创新能力。继续对重点企业实施动态管理，各类信贷资金、企业发展资金、科技3项费用等向重点企业倾斜。突出抓好40户重点企业的58个项目，确保完成投资37亿元，进一步做大做强一批市场潜力大、科技含量高、管理机制活的重点企业。

抓好资源节约工作。抓住国家、省支持资源节约和发展循环经济的机遇，加快实施一批示范价值高、带动能力强、经济效益好的重大项目和示范工程。严格控制高耗能、高耗材、高耗水产业发展，依法关闭破坏资源、污染环境和不符合安全生产条件的企业。全面清理整治砖瓦窑厂，禁止生产实心粘土砖，积极推广新型建材。

（四）进一步加快市区和县域经济发展

突出抓好市区发展。抓住我市被确定为中原城市群核心区的机遇，大力发展二、三产业，密切与郑州的经济联系和功能对接。继续实施经营城市战略，完善机制，深化改革，确保经营城市取得新的成效。以东城区、经济技术开发区、魏都区和许昌县城区为重点，认真搞好项目策划，加快建设步伐，进一步提高市区的辐射带动能力。突出抓好在建城建项目，争取16个续建重点项目年内完成投资13.7亿元。年底前，确保天宝路、许继大道西段、八一路西段、东关大街改造、帝豪游园、滨河游园等项目竣工。加快推进建安公园、曹丞相府及广场工程、107国道市区段改线、许扶运河整治、引佛济许、文昌苑综合开发等项目建设。开工建设许昌至长葛城际通道，带动沿线行政、工业、生态、物流、旅游等项目建设，加快产业聚集，扩大城区规模。

加快发展县域经济。认真贯彻落实全省县域经济工作会议精神，以工业发展为主导，以园区建设为平台，以非公经济为主体，突出特色，深化改革，集约发展，全力推进工业化、城镇化和农业现代化进程。支持和扶植各县（市）大力发展特色经济，推动县域工业相对集中规划和布局，因地制宜发展特色工业园区。加强县（市）城市基础设施和公共服务建设，完善城镇功能，全面提升县域经济发展水平。

深入开展“四城同创”工作。巩固提高“中国优秀旅游城市”、“国家园林城市”创建成果，深入开展“国家文明城市、国家卫生城市、国家森林城市、国家环保模范城市”创建工作。不断完善和规范“组织领导、目标责任、属地管理、资金保障、新闻发布、考核奖惩”的创建机制，让群众参与，让群众受益，让群众满意，切实提高创建水平和实效。

大力发展服务业。以开发三国曹魏文化、钧瓷文化、生态休闲度假等旅游资源为重点，加强旅游重点项目策划和建设，促进旅游业发展。加快发展现代流通和物流业。抓好万里物流园、万通汽贸城、上海商贸城等物流建设项目。加快建设花卉苗木、中药材、发制品、蔬菜、棉花、农机配件等特色物流中心。办好鄢陵花博会、禹州药交会、长葛农机产品交易会和首届国际发制品博览会。积极发展金融、房地产、信息、社区服务等新型服务业。

（五）加大改革开放力度

不断深化企事业改革。完成许继集团、市供水总公司、污水净化公司等企业的产权制度改革，进一步优化股权结构，增强企业发展活力。积极做好东投、交投、发投、城投等市属国有投资公司的改革改制工作，使其真正成为运作规范、盈亏自负、良性发展的市场竞争主体。抓紧推进县（市、区）规模以上集体企业产权制度改革，实现民有民营。力争年内全面完成国有集体企业的产权制度改革。积极推进事业单位改革，巩固完善公益类事业单位“劳动、人事、分配”三项制度改革，加快生产经营型事业单位和准公益类事业单位产权制度改革。

全面推进其它领域的改革。以推进公共财政体系建设为重点，深化改革，扩大覆盖，加强征收和支出管理，增强财政的调控、引导及抗风险能力。以建立和完善政府投资监管体系为重点，深化投融资体制改革。继续做好农村信用社改革。把城市信用社改组为城市商业银行。着力抓好城建、交通、水利等领域的改革。

继续实施开放带动主战略。调整和优化出口商品结构，扩大发制品等传统商品出口，提高机电产品、金刚石制品和高新技术产品出口比重。加快实施“走出去”战略，鼓励发制品、机电、建筑等具有一定实力的企业开展对外投资，带动产品、原材料和技术出口。落实好出口退税和外贸出口推进计划政策，确保出口退税和出口奖励政策及时到位。注重增加先进技术和关键设备的进口，提高企业技术装备水平。

大力发展非公有制经济。继续坚持政治上放心、政策上放开、发展上放手，加强引导、改进服务、优化环境，促进非公有制经济发展壮大。围绕“转机制、抓管理、调结构、增效益”，引导非公有制企业提升内涵。建立完善融资信用担保、维权服务等社会化服务体系，搭建银行与非公有制企业合作、沟通平台，引导金融机构加大对非公有制企业的信贷支持。

（六）加快发展各项社会事业

大力发展科技、教育事业。启动一批重大科技专项和重点项目，集中力量在一些重点领域和关键环节取得突破。继续抓好企业技术中心、工程技术研究中心、重点试验室等创新平台建设，提升我市的自主创新能力和水平。优先发展教育事业，在确保义务教育发展的同时，放开学前教育、高中教育，积极推进职业教育，大力发展民办教育。切实加强农村义务教育，提高农村中小学公用经费标准，建立农村中小学危房改造长效机制，继续对农村义务教育阶段贫困家庭学生实施“两免一补”，加快实施农村中小学远程教育工程。创新人才工作机制，加强人力资源建设。

坚持可持续发展。稳定低生育水平，优化出生人口结构，继续实施农村部分计划生育家庭奖励扶助政策。抓好土地利用总体规划修编，搞好土地资源整理和收储工作，节约集约利用土地。推进矿产资源整合，提高资源开发利用效率。加大环境治理力度，彻底关闭全市19家水泥生产企业的30条普通机械化立窑生产线和禹州市无梁镇小水泥群。

加快文化、卫生、体育、广电等事业和产业发展。按照“盘活存量、激活内力，扩大增量、激活外力”的要求，通过项目带动、招商引资、引入民间资本，实现多元化、多渠道筹资，推动文化、卫生、体育、广电等事业和产业的发展。全面贯彻党的民族宗教政策，依法管理民族宗教事务。认真搞好第二次全国农业普查。继续做好统计、审计、外事、侨务、气象、地震、人防、史志、档案等工作，促进社会事业全面发展。

（七）切实解决关系人民群众切身利益的问题

继续做好就业再就业工作。全面落实就业再就业各项优惠政策，力争新增城镇就业5万人以上，开展再就业培训1.2万人，实现下岗失业人员再就业1.1万人。继续做好大学生就业、复员转业军人安置、城镇新增劳动力和农村富余劳动力就业工作。

进一步完善社会保障体系。巩固企业离退休人员养老金按时足额发放成果，完善养老保险市级统筹工作。以非公有制经济组织和灵活就业人员为重点，继续扩大社会保险覆盖范围。着力解决关闭破产企业退休人员基本医疗保障和困难企业参保问题。完善工伤保险参保办法，抓好事业单位参加工伤保险工作。加强社会救助体系建设，提高城乡居民最低生活保障标准，确保城乡困难群众的基本生活。实施廉租房制度，逐步解决城镇低收入群体住房困难。做好农村医疗救助和长葛市、许昌县、襄城县新型农村医疗合作试点工作，逐步解决农民就医难问题。

整顿和规范市场经济秩序。严厉打击制售假冒伪劣产品、价格欺诈等违法犯罪行为，深入开展假冒商标卷烟等打假治劣专项活动。加强食品药品饮食卫生监管，继续实施食品药品放心工程。加强医疗市场监管，保障人民群众身心健康。规范建筑市场秩序，认真清理建筑领域拖欠工程款和农民工工资，全面实现清欠目标。规范价格秩序，进一步稳定市场物价。

做好安全生产和社会稳定工作。深入开展煤矿、非煤矿山、危险化学品、烟花爆竹、交通运输、建筑施工及消防安全等专项整治，全面落实安全生产责任制、安全生产行政过错责任追究制，坚决遏制重特大事故发生。认真做好人民群众来信来访工作，积极预防和妥善处置群体性事件。强化社会治安综合治理，完善社会治安防控体系，严厉打击各类刑事犯罪和经济犯罪，建设平安许昌。

（八）加强精神文明和民主法制建设

加强精神文明建设。大力倡导以“八荣八耻”为主要内容的社会主义荣辱观，树立知荣明耻、诚实守信的新风尚。广泛开展群众性精神文明创建工作，组织好文化、科技、卫生“三下乡”和科教、文体、法律、卫生“四进社区”活动。加强科普宣传，推进殡葬改革，反对封建迷信，倡导科学文明健康的生活方式。

推进民主法制和国防建设。深入开展普法教育，积极拓展法律服务。认真执行人大及其常委会通过的决定和决议，自觉接受人大及其常委会的法律监督、工作监督和政协的民主监督。认真办理人大代表建议、议案和政协委员提案。加强与民主党派、工商联的联系，发挥工会、共青团、妇联等群众团体作用。全面贯彻落实《依法行政实施纲要》，完善行政决策程序，明确行政执法责任，坚持依法行政。深入开展“双拥”共建活动，继续关心、支持驻许解放军、武警部队和民兵预备役部队建设。

三、关于《许昌市国民经济和社会发展

第十一个五年规划纲要（草案）》的说明

《许昌市国民经济和社会发展第十一个五年规划纲要（草案）》，是在市委的直接领导下研究制定的。《纲要（草案）》力求反映经济社会发展和改革开放新形势的要求，体现宏观性、战略性和政策性，明确政府的工作重点和责任。在《纲要（草案）》的编制过程中，市委、市人大、市政府、市政协多次召开会议，由有关方面组织专家进行咨询论证，在全市范围内开展多种形式的为“十一五”规划建言献策活动，人大代表、政协委员和各界人士提出了许多建议和意见。《许昌市国民经济和社会发展第十一个五年规划纲要（草案）》已提请大会审议。下面，我作简要说明。

（一）“十五”时期国民经济和社会发展主要情况

《纲要（草案）》简要回顾了我市“十五”时期经济社会发展情况。五年来，全市上下紧紧围绕“两个略高、两个确保”目标，深入实施项目带动、招商引资、经营城市、发展非公经济、坚持“两个优先”等重大举措，城乡面貌发生了显著变化，经济社会发展跃上了新的台阶。综合实力显著增强。国民经济连续5年保持了较高的增长速度和质量，生产总值年均增长12.4％，高出全省平均水平，总量比“九五”末翻了一番。人均生产总值达到13411元，与“九五”末相比，增加6771元。地方财政一般预算收入和支出均实现了翻番。县域经济全面发展，5个县（市）综合经济实力均进入全省前40名。城镇化进程明显加快。通过实施经营城市战略和开展创建活动，城市资源得到合理配置，市区、县（市）城区和小城镇协调发展。东城区建设初具规模，许昌市区面积由“九五”末24.5平方公里发展到50.8平方公里，人口由28.5万人发展到51.4万人，全市城镇化率达到32％。“中国优秀旅游城市”、“国家园林城市”创建成功，荣获“河南人居环境奖”、“河南省创建文明城市工作先进城市”称号，创建国家卫生城市通过省级初审。禹州市、长葛市被命名为“中国优秀旅游城市”。基础设施建设成效显著。一批农林水利、交通能源、城市建设、生态环保等基础设施项目建成并投入使用。公路建设累计完成投资70亿元，为“九五”时期的4.6倍。高速公路建设取得突破性进展，“米”字型构架初步形成。县城电网改造全面推进。白沙水库除险加固、东城区水系一期等水利工程顺利竣工。改革开放成果丰硕。国有企业、行政管理、农村税费、粮食流通、财政管理、投资和城市公用事业等体制改革都取得重大进展。对外开放步伐加快，五年累计出口12.8亿美元，是“九五”时期的2.6倍；实际利用外资累计1.5亿美元。人民群众得到较多实惠。城镇居民人均可支配收入年均增长12％，农民人均纯收入年均增长7.6％，分别高出全省2.5和2.6个百分点。养老、医疗、失业、工伤、生育保险和城乡居民最低生活保障制度相继建立，城乡社会救助体系逐步完善。社会事业全面进步。科技、教育、文化、卫生、体育、广电等事业协调发展。许昌师专升格为许昌学院，筹建了许昌职业技术学院。荣获“全国双拥模范城”、“全国科技进步先进市”称号。连续2年被评为“全省社会治安综合治理先进市”。社会主义政治建设、文化建设、社会建设取得新进展。“十五”时期取得的显著成绩，极大地增强了全市人民加快发展、科学发展的信心。

（二）“十一五”时期经济社会发展的总体思路和发展目标

根据党的十六届五中全会、省委七届十次全会和市委四届六次全会精神，结合我市实际，《纲要（草案）》提出了我市“十一五”时期经济社会发展的总体思路、总体原则和发展目标。

在总体思路上，提出了坚持以科学发展观统领经济社会发展全局。《纲要（草案）》以加快发展、科学发展为主线，注重对产业发展的科学谋划，提出了创新体制，创优环境，建设资源节约型、环境友好型社会，促进社会和谐的思路。《纲要（草案）》总结了“十五”时期我市经济社会发展的经验，把大力发展非公有制经济和“两个优先”写入了总体思路，体现了工作的连续性，也符合我市实际。

在总体原则上，提出了“六个正确处理、六个注重”。即：正确处理加快发展和科学发展的关系，注重在加快发展中解决前进中的矛盾和问题；正确处理结构、速度、质量、效益的关系，注重结构调整和转变经济增长方式，提高经济运行的质量和效益；正确处理中心城市带动、县域经济发展与建设社会主义新农村的关系，注重加快市区经济发展和城乡互动；正确处理启动内在活力和借助外力加快发展的关系，注重把体制创新与对外开放作为经济社会发展的原动力；正确处理经济发展与人口、资源、环境协调统一的关系，注重可持续发展，建设资源节约型、环境友好型社会，使经济社会发展步入良性轨道；正确处理经济建设与社会事业协调发展的关系，注重坚持以人为本、改善群众生活、构建和谐社会。这是落实科学发展观的具体要求，是保持经济社会快速健康协调发展必须坚持的原则。

在主要目标上，提出了生产总值年均增长12%。这是综合考虑各方面因素，根据需要和可能提出的，既与全面建设小康社会的目标相衔接，又反映了经济社会发展的阶段性特征和客观要求。这个目标比全国、全省提出的目标略高了一些，是积极的，是经过努力可以实现的。需要说明的是，《纲要（草案）》提出财政一般预算收入要在2005年基础上实现翻一番，单位生产总值能耗5年降低20％。这两个指标是反映经济增长质量和经济转型的重要指标，要在确保完成的前提下，争取更大成效。

（三）“十一五”时期的主要任务

一是以工业化为重点推进产业结构优化升级。《纲要（草案）》提出，要大力推进“136”工业体系建设。以许继高科技电气城为依托，加快国家（许昌）电力电子系统产业园建设。通过政策扶持、体制创新、招商引资等多种形式，巩固发展烟草工业、金刚石生产、发制品生产3大基地。充分发挥比较优势，培育壮大食品工业、汽车及零部件制造业、纺织服饰业、造纸工业、能源工业、新型建材业等6大优势产业。加快产品结构升级，培育一批科技含量高、成长性强的大企业集团。壮大许昌经济技术开发区、河南（魏都）民营科技园区、许昌县尚集民营工业园，以及禹州市、长葛市、鄢陵县、襄城县等工业园区规模，发展产业集群。《纲要（草案）》提出，要加快发展服务业，特别是旅游、物流、房地产、金融、保险和社区服务业，不断提高服务业的比重和水平，进一步优化经济结构。加快推进信息化，努力建设数字许昌。

二是以农业产业化为重点建设社会主义新农村。《纲要（草案）》提出，要积极推进社会主义新农村建设，重点建设许昌至长葛城乡一体化新农村示范区。加快花卉苗木、中药材、烟叶等特色农业基地建设和畜禽业发展。发挥国家农业科技园区作用，积极推进优质农产品生产基地、高技术企业孵化基地、新型农业人才培训基地和现代农业示范基地建设。加大对重点农产品加工企业的扶持力度，形成一批技术装备水平高、带动能力强、具有持续发展能力的农业产业化龙头企业。以建立和完善市、县、乡三级农业信息服务平台为主攻方向，建设国家农业信息化示范市。

三是以城镇化为重点推动区域协调发展。《纲要（草案）》从我市的实际出发，按照中原城市群发展的要求，明确了我市城市定位和发展思路。《纲要（草案）》提出，要进一步完善城市规划和产业布局，以许昌至长葛城际通道为轴线，积极推进示范区城乡一体化发展，形成带状城市。充分发挥东城区在市区发展中的重要带动作用，把东城区建设成为综合服务功能完善、外向型经济充分发展的现代新城区。强化城市干道与国道主干网和高速公路网的连接，进一步拉大城市框架。深入开展“四城同创”活动，提升城市管理水平。加强县（市）城区供水、污水和垃圾处理等基础设施建设，不断完善城市功能。

四是建设资源节约型、环境友好型社会。《纲要（草案）》提出，要构建煤及煤化工、电力、水泥、中水回用、废旧有色金属加工、固体废料利用等六大产业循环链，发展工业循环经济；抓好绿色无公害种植和养殖以及农畜产品的精深加工与综合利用，发展农业循环经济。加强生态建设与环境保护，创建国家环保模范城市。大力推进节能、节水、节材、节地和资源的综合利用，建设节约型城市。

五是进一步深化改革和扩大开放。《纲要（草案）》提出，要深化国有企业、国有资产管理体制和集体企业改革。加快公用事业改革，积极推进事业单位改革。重点推进行政管理体制改革。加大招商引资力度，拓宽引资渠道，努力提高利用外资的规模和质量。加强与东部沿海发达地区的合作，积极承接东部地区产业转移，引进一批项目和资金。加大出口商品结构调整力度，提高技术密集型、资本密集型产品的出口比重。

六是加快科技、教育、文化、旅游等事业发展。《纲要（草案）》提出，要促进自主创新，建立健全技术创新体系、科技服务体系和技术推广体系，走以企业为主体、市场为导向、产学研相结合的技术创新之路。围绕我市具有产业基础和技术优势的重点领域，大力发展高新技术产业和民营科技企业。巩固和提高义务教育，大力发展职业教育和民办教育，不断提高高等教育质量。积极推进文化、卫生、体育、广电、旅游等事业单位改革，走事业、产业共同发展的路子。坚定不移地走人才强市之路，努力营造人才辈出、人尽其才、才尽其用的社会氛围。

七是加强和谐许昌建设。《纲要（草案）》提出，要实施积极的就业政策，创造更多就业岗位。完善养老、医疗、失业、工伤、生育等保险制度，扩大覆盖面，提高保障能力。高度重视安全生产工作，加大安全生产投入，健全安全生产监管体制。强化药品、食品、餐饮卫生等市场监管，保证人民群众健康安全。做好人口和计划生育工作。加强社会治安综合治理，维护社会稳定。加强社会主义政治文明和精神文明建设。

各位代表！

全面完成今年和“十一五”期间的任务，对各级政府提出了更高的要求。

我们要进一步转变政府职能。大力推进政府管理创新，注重社会管理和公共服务，努力建设高效、法治、服务、廉洁政府。继续推进政务公开，不断深化行政审批制度改革。建立健全社会预警体系和应急救援机制，提高处置突发事件和保障公共安全的能力。坚持以人为本，办好“10件实事”。注重质量效益，促进社会和谐，完善绩效和目标管理考评办法，实行每季度一次的加快发展科学发展新闻发布会制度。

我们要进一步加强廉政建设。认真落实廉政建设责任制，集中开展治理商业贿赂专项工作，重点治理工程建设、土地出让、产权交易、医药购销和政府采购等领域的问题。建立健全经济责任审计制度。加强行政效能监察，严厉查处滥用权力、以权谋私等违纪违法案件，坚决纠正损害群众利益的不正之风，促进政府工作人员廉洁从政。

我们要进一步提升政府效能。认真实施公务员法，加强对公务员的教育、管理和监督。按照“狠抓落实年”的要求，继续推行“政务公开制、首位负责制、服务承诺制、限时办结制、责任追究制”，着力抓好提高执行力、整治机关病、强化案件查处、改善企业周边环境等四项重点工作，促进政府效能提升。坚持把加快发展、科学发展作为第一要务，把当好公仆、为民办事作为第一职责，把狠抓落实、苦干实干作为第一选择，求真务实，艰苦奋斗，多做打基础、利长远、促发展、惠民众的事情。

各位代表!

“十五”时期取得的巨大成就，为我们奠定了良好的发展基础；国家和省促进中部地区崛起、建设社会主义新农村的战略举措，为我们提供了难得的历史机遇；“十一五”规划的制定实施，为我们绘就了未来的美好宏图。让我们在中共许昌市委的正确领导下，紧紧依靠全市人民，以更加饱满的热情、昂扬的斗志、创新的精神、务实的作风，抓住机遇，加快发展，努力把“十一五”规划的宏伟蓝图变为美好现实，在全面建设小康社会的伟大实践中谱写出崭新的篇章！
